# Whole-genome and targeted sequencing of drug-resistant *Mycobacterium tuberculosis* on the iSeq100 and MiSeq: A performance, ease-of-use, and cost evaluation

**DOI:** 10.1371/journal.pmed.1002794

**Published:** 2019-04-30

**Authors:** Rebecca E. Colman, Aurélien Mace, Marva Seifert, Jonathan Hetzel, Haifa Mshaiel, Anita Suresh, Darrin Lemmer, David M. Engelthaler, Donald G. Catanzaro, Amanda G. Young, Claudia M. Denkinger, Timothy C. Rodwell

**Affiliations:** 1 Foundation for Innovative New Diagnostics, Campus Biotech, Geneva, Switzerland; 2 Department of Medicine, University of California, San Diego, San Diego, California, United States of America; 3 Illumina Inc., San Diego, California, United States of America; 4 Translational Genomics Research Institute, Flagstaff, Arizona, United States of America; 5 Department of Biological Sciences, University of Arkansas, Fayetteville, Arkansas, United States of America; John Hopkins University, UNITED STATES

## Abstract

**Background:**

Accurate, comprehensive, and timely detection of drug-resistant tuberculosis (TB) is essential to inform patient treatment and enable public health surveillance. This is crucial for effective control of TB globally. Whole-genome sequencing (WGS) and targeted next-generation sequencing (NGS) approaches have potential as rapid in vitro diagnostics (IVDs), but the complexity of workflows, interpretation of results, high costs, and vulnerability of instrumentation have been barriers to broad uptake outside of reference laboratories, especially in low- and middle-income countries. A new, solid-state, tabletop sequencing instrument, Illumina iSeq100, has the potential to decentralize NGS for individual patient care.

**Methods and findings:**

In this study, we evaluated WGS and targeted NGS for TB on both the new iSeq100 and the widely used MiSeq (both manufactured by Illumina) and compared sequencing performance, costs, and usability. We utilized DNA libraries produced from *Mycobacterium tuberculosis* clinical isolates for the evaluation. We conducted WGS on three strains and observed equivalent uniform genome coverage with both platforms and found the depth of coverage obtained was consistent with the expected data output. Utilizing the standardized, cloud-based ReSeqTB bioinformatics pipeline for variant analysis, we found the two platforms to have 94.0% (CI 93.1%–94.8%) agreement, in comparison to 97.6% (CI 97%–98.1%) agreement for the same libraries on two MiSeq instruments. For the targeted NGS approach, 46 *M*. *tuberculosis*–specific amplicon libraries had 99.6% (CI 98.0%–99.9%) agreement between the iSeq100 and MiSeq data sets in drug resistance–associated SNPs. The upfront capital costs are almost 5-fold lower for the iSeq100 ($19,900 USD) platform in comparison to the MiSeq ($99,000 USD); however, because of difference in the batching capabilities, the price per sample for WGS was higher on the iSeq100. For WGS of *M*. *tuberculosis* at the minimum depth of coverage of 30x, the cost per sample on the iSeq100 was $69.44 USD versus $28.21 USD on the MiSeq, assuming a 2 × 150 bp run on a v3 kit. In terms of ease of use, the sequencing workflow of iSeq100 has been optimized to only require 27 minutes total of hands-on time pre- and post-run, and the maintenance is simplified by a single-use cartridge–based fluidic system. As these are the first sequencing attempts on the iSeq100 for *M*. *tuberculosis*, the sequencing pool loading concentration still needs optimization, which will affect sequencing error and depth of coverage. Additionally, the costs are based on current equipment and reagent costs, which are subject to change.

**Conclusions:**

The iSeq100 instrument is capable of running existing TB WGS and targeted NGS library preparations with comparable accuracy to the MiSeq. The iSeq100 has reduced sequencing workflow hands-on time and is able to deliver sequencing results in <24 hours. Reduced capital and maintenance costs and lower-throughput capabilities also give the iSeq100 an advantage over MiSeq in settings of individualized care but not in high-throughput settings such as reference laboratories, where sample batching can be optimized to minimize cost at the expense of workflow complexity and time.

## Abstract 2

Rebecca E. Colman and colleagues assess performance, cost, and throughput of a new sequencer designed for detecting drug-resistant tuberculosis strains.

## Author summary

**Why was this study done?:**

- *M*. *tuberculosis* is the leading cause of death from a single infectious agent.
- Diagnosis of drug-resistant *M*. *tuberculosis* is imperative for reducing tuberculosis (TB) mortality.
- Next-generation sequencing (NGS) can provide comprehensive genetic information on drug resistance for supporting rapid clinical decision-making but currently can only be performed at the reference-laboratory level.
- Illumina’s solid-state benchtop sequencer iSeq100 adds diversity to the intended use settings in which NGS can be practically utilized for drug-resistant TB detection, but it has not yet been demonstrated that existing TB NGS workflows will perform on the new instrument.

**What did the researchers do and find?:**

- The study was focused on the performance of existing TB whole-genome sequencing (WGS) and targeted NGS approaches that work on the Illumina MiSeq to determine if they could be also utilized on the iSeq100 platforms.
- WGS library preparations were performed on three strains, and the same libraries were run on two MiSeq instruments and one early-access iSeq100 instrument. The percentage of genome covered was similar across the instruments and the three isolates, ranging from 99.20% to 99.92% of the genome covered.
- Targeted NGS preparations were performed on 46 strains and run on two MiSeq instruments and one iSeq100 instrument with 99.6% (CI. 98.0%–99.9%) agreement on drug resistance–associated SNPs between iSeq100 and MiSeq data sets.
- Because of batching-based pricing constraints for TB sequencing, the sequencing cost per sample is higher on the iSeq100 platform than for the same coverage on the MiSeq when batched optimally. However, the iSeq100 allows for a more open-access workflow due to a lower number of samples being in one sequencing batch. This relationship is the same for WGS and targeted NGS, although the scale is different depending on the sequencing approach chosen.

**What do these findings mean?:**

- In settings of individualized TB care, lower throughput will drive a different need for batching, and the iSeq100, with its lower-throughput capabilities, has an advantage over MiSeq. However, in high-throughput settings such as reference laboratories, where sample batching can be optimized to minimize cost at the expense of workflow complexity and time, the MiSeq will make the most sense.

## Introduction

Tuberculosis (TB) mortality has been declining at a rate of approximately 3% per year since 2000 [1]. Although encouraging, this trajectory will not lead to achieving the End TB target of a 95% reduction of TB deaths by 2035 [2]. Given that drug-resistant TB (DR-TB) is a key driving factor behind TB mortality globally, implementation of comprehensive, rapid drug susceptibility testing (DST) is critical in all settings. Detection of resistance-conferring mutations by molecular methods is a rapid and accurate alternative to phenotypic DST and has been shown to provide actionable information to healthcare workers (e.g., GeneXpert MTB/RIF) [1]. Currently, molecular resistance tests provide only partial data on drug susceptibility and are limited by the number of gene targets examined. With the introduction of novel and repurposed drugs, new treatment regimens, and increasing numbers of patients with complex resistance profiles, a more comprehensive solution for guiding patient treatment becomes crucial. The rapid evolution of knowledge on the genetic determinants of TB drug resistance suggests that gene sequencing will become the most appropriate and versatile technology platform to provide rapid, accurate, and actionable results for treatment of this disease [3,4]. A next-generation sequencing (NGS) approach gives comprehensive genetic information on drug resistance–related genes, be it through whole-genome sequencing (WGS) or targeted NGS [5].

Although NGS can be used to answer a multitude of questions in TB, spanning patient care, drug resistance surveillance, and contact tracing, each of these questions have specific requirements for laboratory processing and implementation, particularly in low- and middle-income countries (LMICs). NGS instrumentation is increasingly found in more clinical laboratories; however, it has primarily been driven by human genomics applications and has only recently begun to be used for infectious disease applications [6] and thus needs to be extended to LMICs [5,7]. For successful use in LMICs, a sequencer must be robust, easy to use, and affordable. Illumina instruments are the most widely deployed sequencers currently being used for TB and infectious disease work [5,8]. However, the MiSeq instrument’s price point, maintenance requirements, and complexity make it better suited for reference laboratory use in comparison to a more decentralized solution. The newly released Illumina iSeq100 was designed with the goal of a simplified workflow, lower capital investment of the instrument, increased instrument robustness, and reduced maintenance requirements [9], potentially allowing expansion of sequencing solutions to more healthcare facilities and resulting in a more decentralized solution.

We examined how the Illumina sequencing platform iSeq100 fits into the workflows already being developed. To this end, we ran both WGS and targeted NGS laboratory workflows for *M*. *tuberculosis* on the Illumina iSeq100 instrument alongside the MiSeq instrument. Using isolate DNA from clinical samples, we examined the usability of the sequencing instrument for both WGS and targeted NGS for drug resistance detection in *M*. *tuberculosis*.

## Methods

### Performance

#### WGS

Three *M*. *tuberculosis* isolates were selected from a well-characterized archive collection at the University of California, San Diego (UCSD) [10,11], for WGS. Nextera DNA flex libraries were prepared following the manufacturer’s protocol [12]. A total of 10 ng of DNA was used as the input into the library preparation, and 12 PCR cycles were run, resulting in dual-indexed final libraries. Libraries were pooled in equal molar concentrations, and the same libraries were sequenced on each instrument. Sequencing on the MiSeq platform using MiSeq Reagent kit v3 (Illumina) and paired-end 2 × 300 bp sequencing was performed both at the Institute for Genomic Medicine (IGM) Genomics Center at UCSD and at Illumina, San Diego. Sequencing on iSeq100 was performed at Illumina on an early-access version of the platform with a 2 × 150 bp run. Three isolates were pooled together for the goal of approximately 100× coverage on the iSeq100 platform based on 2 × 150 bp run and a genome size of 4.412 Mb. Each whole-genome pool was spiked with approximately 2% of PhiX sequencing control prior to sequencing. The iSeq100 run was loaded at 50 pM and, because of the low complexity of the run, was demultiplexed with a single index as per suggestion of Illumina after demultiplex analysis. FASTQ files were run through an Amazon cloud version of the ReSeqTB platform unified variant analysis pipeline [13] for WGS, and variant calls (SNPs and insertion/deletions) were compared across sequencing instruments.

#### Targeted sequencing

Forty-six *M*. *tuberculosis* isolates were selected from the same archive collection at UCSD [10,11] for the targeted NGS work. Targeted libraries were prepared following an optimized protocol based on the previously published Next-Gen RDST approach [14,15] focused on six genomic regions (*inhA* promoter, *katG*, *rpoB*, *gyrA*, *eis* promoter, and *rrs*) conferring resistance to seven target drugs: isoniazid (INH), rifampin (RIF), moxifloxacin (MOX), ofloxacin (OFX), amikacin (AMK), kanamycin (KAN), and capreomycin (CAP). Because of the limited availability of indexed primers, two pools of 26 libraries were made for the MiSeq runs instead of a single pool. With the lower data output produced on the iSeq100, the samples were split across five pools containing either 10 or 11 samples. The sequencing methods used were the same as with WGS but spiked with 25% and 2% of PhiX sequencing control for the MiSeq and iSeq100 runs, respectively. With only 26 samples per pool for the MiSeq runs, the pool loading concentration was not run at maximum capacity because of the lower number of samples on the run than optimal. The targeted NGS protocol used in this study requires custom sequencing primers, which is a feature on the commercially available MiSeq reagent cartridge but not yet a feature on the commercially available iSeq100. For the MiSeq runs, specific read 1, read 2, and indexing sequencing primers were used for sequencing and were added for a final concentration of 0.5 μM [14]. On the iSeq100, these same custom primers were added, with the only difference being that the reverse complement of indexing sequencing primer used on the MiSeq was needed for the iSeq100. For iSeq sequencing of the targeted NGS libraries in this study, a development prototype cartridge that is not commercially available was used by Illumina. FASTQ files were run through an optimized TB-ASAP analysis pipeline [15] that is currently being developed to be added into the ReSeqTB platform for targeted NGS analysis. High-confidence drug resistance–associated SNP [3] calls were compared across sequencing instruments.

The prospective protocol is provided in S1 Text. All analyses done through the ReSeqTB platform were performed by FIND and UCSD (S2 Text). The iSeq100 pool loading concentrations were determined by analysis done at Illumina.

#### Nucleotide sequence accession numbers

All sequencing read files were deposited in the NIH Sequence Read Archive (http://www.ncbi.nlm.nih.gov/bioproject/505787) under BioProject no. PRJNA505787.

### Cost/batching

All cost analysis was based on United States list pricing as of October 2018. For the different batching schemes for WGS, the minimum average depth of coverage was set to approximately 30× and was calculated with the assumption of *M*. *tuberculosis* genome size of 4.412 Mb. The MiSeq system has four versions of sequencing kits that were used for the batching and cost estimate, both using 2 × 150 bp run setup for a direct comparison to the iSeq100. The batching schemes for targeted NGS were calculated based on the total number of reads expected from each sequencing platform, and assuming a six-amplicon targeted NGS approach, using only the MiSeq 2 × 300 bp sequencing kit.

### Instrumentation/ease of use

System guides for both sequencing instruments were used to calculate the number of steps and hands-on time needed to start a sequencing run [9, 16]. Additionally, information from publicly available fact sheets was used for comparison of specific characteristics of the MiSeq and the iSeq100.

## Results

### Performance

#### WGS

**Fig 1:**
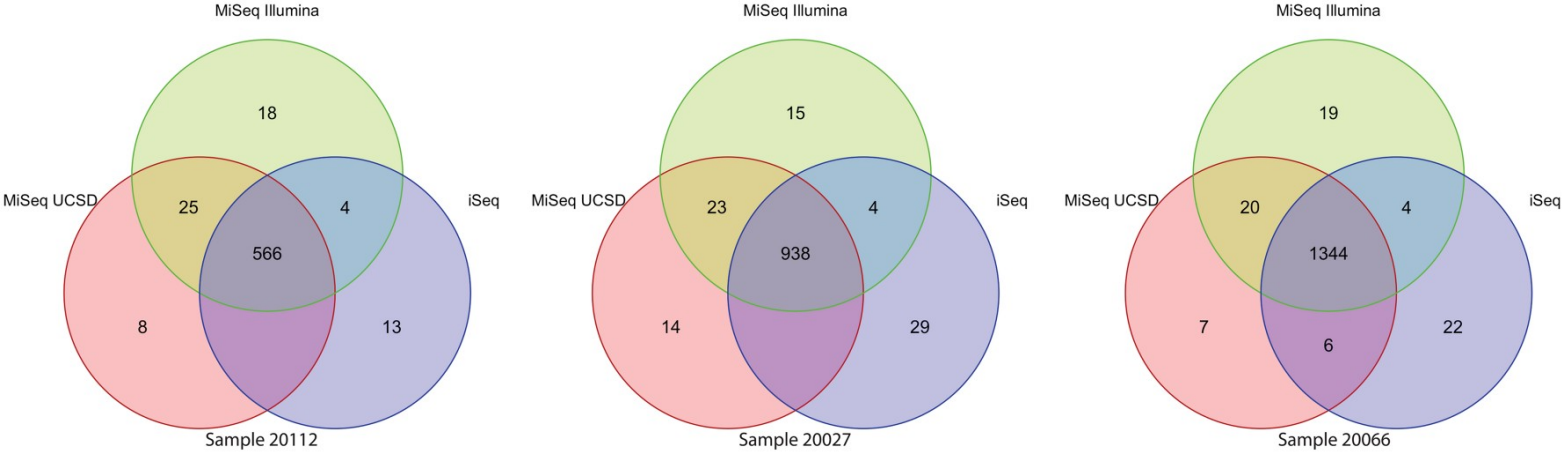
Comparison of WGS variant calls. Comparison of variant calls for individual Nextera DNAFlex WGS libraries sequenced on the MiSeq run at Illumina, the MiSeq run at UCSD, and the iSeq100 (green, red, and blue, respectively). Variants were identified using the ReSeqTB UVP. UCSD, University of California, San Diego; UVP, unified variant pipeline; WGS, whole-genome sequencing.

Average depth of coverage for the three samples across all three sequencers was consistent with sequencer specifications (Table 1). Difference in coverage between the MiSeq run at Illumina and the MiSeq run at UCSD was due to loading concentration differences between the two runs. The coverage was uniform across the genome for all three libraries with no difference between the sequencers. The variant call percent agreements were 91.9%, 93.1%, and 96.2% for sample ID 20112, 20027, and 20066, respectively (Fig 1, S1 Table), for the MiSeq run at UCSD and the iSeq100 data. The average variant call percent agreements were 97.6% (CI 97%–98.1%), 94% (CI 93.1%–94.8%), and 93.6% (CI 92.6%–94.4%) for MiSeq at Illumina/MiSeq at UCSD, MiSeq at UCSD/iSeq100, and MiSeq at Illumina/iSeq100, respectively (S1 Table).

**Table 1:** WGS results.

Sequencer and Location	Sequencing Characteristics	20112	20027	20066
MiSeq–UCSD	Depth of coverage	621×	1,084×	490×
	Genome breadth (%)	99.86	99.92	99.57
	Number of reads	22,266,902	16,477,974	38,822,318
MiSeq–Illumina	Depth of coverage	397×	831×	360×
	Genome breadth (%)	99.77	99.87	99.20
	Number of reads	10,904,713	9,647,180	24,251,807
iSeq100	Depth of coverage	81×	149×	65×
	Genome breadth (%)	99.77	99.86	99.22
	Number of reads	3,965,793	3,169,624	7,449,903

#### Targeted sequencing

All 46 samples were sequenced with high average depths of coverage of 45,263×, 68,786×, and 67,792× across six genomic targets for the MiSeq run at Illumina, MiSeq run at UCSD, and the iSeq100 run, respectively. Read 2 was not used for the iSeq100 analysis because of underperformance related to the custom sequencing primer. We compared six gene targets for the 46 samples, resulting in 276 comparisons. For the targeted NGS work, the drug resistance–associated SNP call percent agreement was 100% between the MiSeq runs and 99.6% between the MiSeq and iSeq100 runs. The only difference that occurred was due to a low-percentage SNP call on the iSeq (6% resistant allele, 94% susceptible allele) that was below calling threshold on the MiSeq (2% resistant allele, 98% susceptible allele) for a single sample.

### Cost/batching description

**Fig 2:**
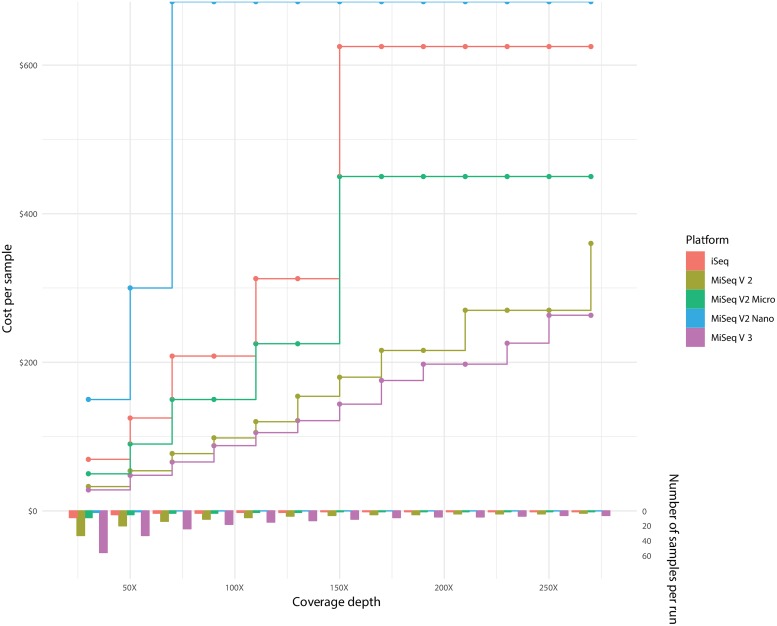
Cost per sample with varying batching schemes for MiSeq and iSeq platforms. WGS depth of coverage calculated assuming *M*. *tuberculosis* genome and 2 × 150 bp runs. Minimum of 30× coverage was used for all scenarios. The bar graph depicts the batching size for the different scenarios. Red, brown, green, blue, and purple data depict the iSeq100, MiSeq version 2 kit, MiSeq version 2 Micro kit, MiSeq version 2 Nano kit, and MiSeq version 3 kit, respectively. Prices are based on list price as of October 2018 and are in USD. WGS, whole-genome sequencing.

**Fig 3:**
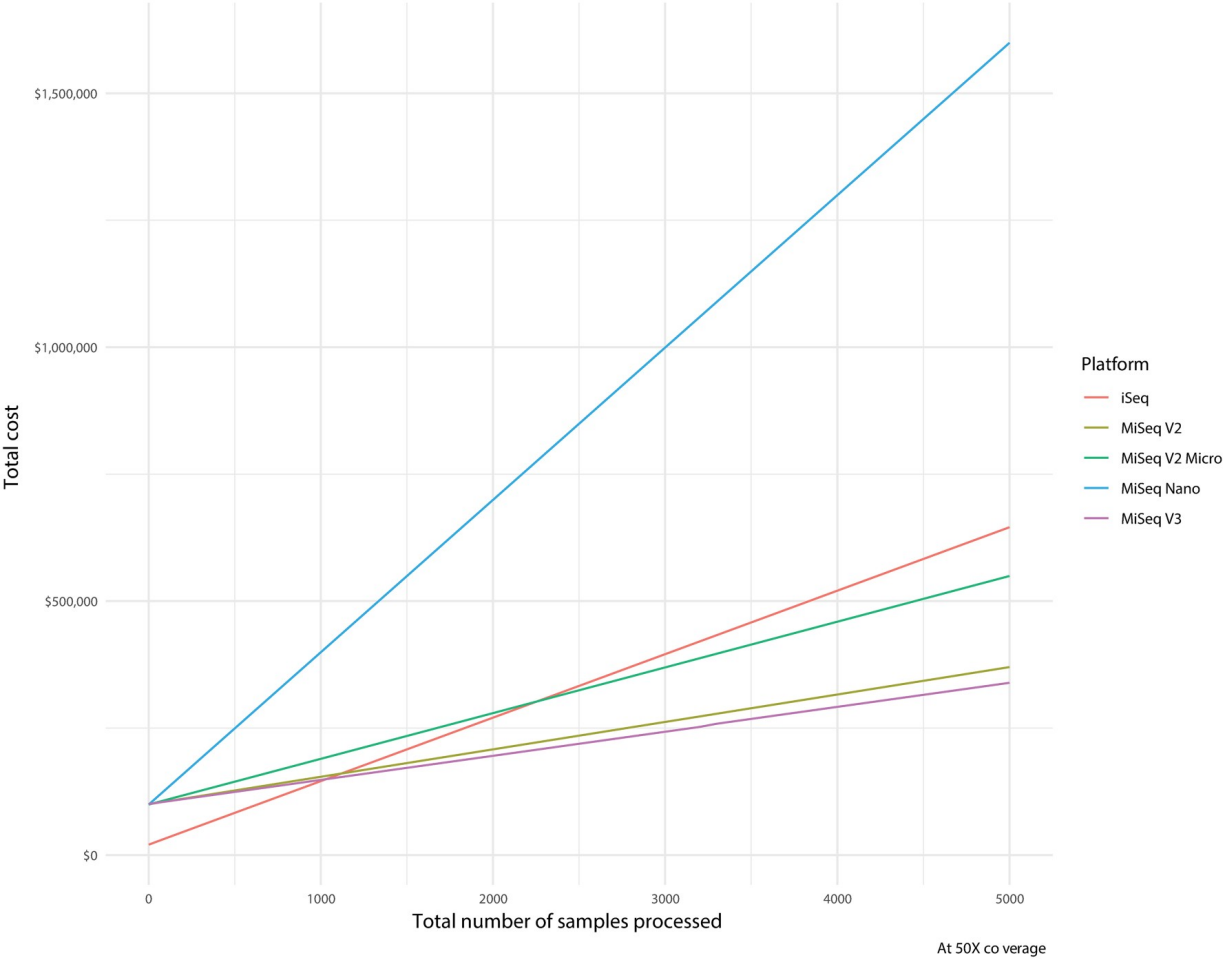
Cost trajectories with total numbers of samples processed. Cost per sample was calculated for average depth of 50× coverage using 2 × 150 bp runs. Total cost includes capital cost of sequencer and sequencing cost per sample. Red, brown, green, blue, and purple data depict the iSeq100, MiSeq version 2 kit, MiSeq version 2 Micro kit, MiSeq version 2 Nano kit, and MiSeq version 3 kit, respectively.

The MiSeq and iSeq100 have listed instrument prices of $99,000 USD and $19,900 USD, respectively. The difference in sequencing cost per sample for WGS with varying batching sizes and resulting depth of coverage can be found in Fig 2. Combining the capital costs for the sequencing instrument and sequencing cost per sample, cost trajectories were calculated for different sequencing kits (Fig 3). For targeted NGS, the relationship of cost per sample, batching sizes, and resulting depth of coverage will be the same as with WGS, but the scale and details will be different. The difference in sequencing cost per sample for this targeted NGS approach was calculated based on the total number of reads expected from each sequencing platform/kit and assuming a six-amplicon targeted NGS approach (Table 2).

**Table 2:** Targeted NGS batching effect on cost per sample.

iSeq100			MiSeq v3 kit		
Depth of coverage (x)^a^	Cost per sample (USD)	Number of samples	Depth of coverage (x)^a^	Cost per sample (USD)	Number of samples
60,606	$56.82	11	160,256	$60.77	26
30,303	$28.41	22	60,386	$22.90	69
15,152	$14.20	44	30,864	$11.70	135
10,101	$9.47	66	15,152	$5.75	275

^a^Depth of coverage based on total number of reads expected for each sequencing system and a six-amplicon targeted NGS approach.

Abbreviation: NGS, next-generation sequencing.

### Instrumentation description/ease of use

The iSeq100 has a smaller instrument footprint than the current MiSeq instrument (Table 3). For sequencing reagents, currently the iSeq100 has one kit available that can sequence up to 2 × 150 bp runs, and the MiSeq sequencer has two options for sequencing reagents—reagent kit v2 and reagent kit v3—each with different options for the number of sequencing cycles. Both fluidics and imaging components are built into the consumable on the iSeq100, resulting in reduction of maintenance required and number of steps to start a sequencing run (Table 3).

**Table 3:** Instrumentation comparison of MiSeq and iSeq100 platforms.

Characteristic	MiSeq	iSeq100
Footprint (cm) W × H × D	68.6 × 52.3 × 56.6	42.5 × 30.5 × 33
Number of steps to start sequencing^a^	74	59
Number of steps after sequencing run^a^	27	6
Maintenance	Washes monthly, standby wash after idle mode (idle for 7 days)	Air filters every 6 months
Hands-on time^b^	Approximately 53 minutes	Approximately 27 minutes
Time to completion (2 × 150 bp run)	24 hours	17.5 hours

^a^Calculated from the system guides.

^b^Calculated including both setup and post-run steps.

## Discussion

Adoption of NGS to examine a multitude of questions in TB research has rapidly occurred over the past few years. The use of NGS for comprehensive drug resistance profiles through examination of drug resistance–associated mutations is a promising tool for clinical care. Moving NGS workflows into clinical settings from research settings will require an evaluation of the performance of established workflows on different sequencing instruments, possibly opening new avenues for establishing NGS for TB clinical use. In this study, the iSeq100 platform’s performance was comparable to the commonly used MiSeq platform (Fig 1, S1 Table), illustrating the potential interchangeability of different sequencers in a laboratory setting.

In the research setting, WGS is currently used to understand different aspects of clinically important questions, such as drug resistance profiles for patient care, transmission mapping, and drug resistance surveillance [4,5,17]. The iSeq100 performance on WGS was comparable in regard to genome breadth of coverage with above 99% for all samples, regardless of sequencing platform (Table 1). Using the ReSeqTB analysis pipeline for both MiSeq data sets and the iSeq100 data set allowed for comparison of variant call differences, both SNPs and insertion/deletions, that were driven by sequencer differences and not pipeline differences. Overall, variant calls were highly concordant across samples and between sequencers (Fig 1). Differences in variant calls between sequencers could be due to the difference in optics between the two systems. The 13, 29, and 22 variant calls (Fig 1) found only in the iSeq100 data for samples 20112, 20027, and 20066, respectively, and the 51, 52, and 46 variant calls only found from the MiSeq data for samples 20112, 20027, and 20066, respectively, are likely due to the difference in dye chemistry and clustering efficiency between the platforms. However, it is important to note that random sequencing error, clustering density, percentage of reads passing the filter, and the overall noise in the sequencing signal resulted in 26, 29, and 26 variant calls when comparing the MiSeq run at UCSD and the MiSeq run at Illumina for samples 20112, 20027, and 20066, respectively (Fig 1). The WGS results demonstrate the ability to take an existing workflow and utilize the solid-state sequencing instrument by only changing the number of samples pooled together. Further optimization of the pool loading concentration and evaluation of different index pooling schemes will be needed to fully utilize the iSeq100 platform for an optimized amount of data output.

For high-income countries with a low TB burden, the reliance on culture as input for sequencing allows for tailored solutions for their unique needs, but this is likely unsuited for the introduction and uptake of NGS in LMICs. For the effective increase in characterization of drug resistance worldwide, the ability to conduct sequencing on patient samples directly without the need for culture will be critical for both patient care and surveillance. A targeted sequencing approach allows for workflows to be developed and implemented in areas without culturing facilities. The targeted NGS results had a high percentage of agreement when examining drug resistance–associated SNPs, again demonstrating the ability to take existing laboratory workflows and implement a simpler sequencer for the final step. Depending on the targeted sequencing approach used, the read lengths that are available on each sequencing platform need to be taken into account. Ensuring that the loci of interest will be captured in the read length that is selected for the MiSeq (up to 300 bp) or for the iSeq (up to 150), with this assay used in this study all mutations of interest are covered by a 150 bp sequencing read. One complexity of using the early-access iSeq100 instrument is that the targeted NGS approach used in this study requires the addition of sequencing primers not found in the Illumina reagent cartridge, and this addition of primers is currently not supported in the released reagent cartridge. For this study, Illumina provided this capability on a development prototype cartridge, but optimization is needed for successful sequencing of read 2. The read 2 data were of poor quality and did not align to the reference; however, even without using read 2 data, the iSeq100 drug resistance–associated SNPs had a high level of agreement (99.6% CI 98.0%–99.9%) with the MiSeq SNP calls. Not all targeted NGS approaches need these additional primers, and thus the sequencing would transition in the same way as the WGS workflows onto the iSeq100 platform. Future iterations of the instrument/cartridges for the iSeq100 may allow for the addition of other sequencing primers. By ignoring read 2 data for the prototype iSeq100 cartridge, we were able to show a high performance of the targeted approach on both instruments. In 276 SNP comparisons, we found only one difference between the iSeq100 and the MiSeq platforms. This difference was driven by a low-level mixture that was above the 5% threshold on the iSeq100 and below the threshold on the MiSeq. Further work exploring the error profiles for each platform will inform the setting of thresholds on low-level mixtures.

Molecular technologies including NGS are having a significant impact on the diagnosis of infectious disease. However, for a greater impact, sequencing technology needs to be relatively low cost. A key benefit to an NGS approach to drug resistance detection is the scalable and flexible throughput. The reduced data output of the iSeq100 [9] allows for smaller batch sizes without using excess sequencing space. Smaller batch sizes allow for more open-access workflows, decreasing the time to result for patient samples versus needing to wait until batch size is met before processing. However, there is a trade-off to the iSeq100’s reduced batch size: the reagent cost per sample will be higher than on instruments for which price reduction is driven by a high degree of batching samples. For WGS at the highest batching levels to achieve roughly 30× coverage, the cost per sample on the iSeq100 is $69.44 USD, going down to $28.21 USD for the comparable 2 × 150 bp run on a MiSeq and reducing even further to $13.98 USD if run on a 2 × 300 bp run (Fig 2), excluding the cost of the sequencing instrument, personnel time, and instrument maintenance. To achieve the cost of $28.21 USD for the sequencing run, 56 WGS samples have to be batched together on the MiSeq 2 × 150 bp run versus only 9 samples batched together on the iSeq100 (Fig 2). Additionally, there are nano and micro versions of the MiSeq reagent kits that also reduce data output and could be used in different batching scenarios (Fig 2). The same relationship between sequencing costs per sample, number of samples per batch, and depth of coverage is found for targeted NGS, resulting in more samples per batch and lowering the cost per sample. For this targeted NGS solution, at the highest batching to levels to achieve about 15,000× coverage, the sequencing cost per sample goes from $14.20 USD on the iSeq100 down to $5.75 USD on the MiSeq v3 kit (Table 2), excluding the cost of the sequencing instrument, personnel time, and instrument maintenance. However, to achieve this, 44 samples must be batched on the iSeq100 versus 135 samples on the MiSeq. Individualized care and the resulting throughput is unlikely to maximize the batching of the higher-throughput sequencers like the MiSeq, which increase the time to answer depending on the waiting time to get the minimum number of samples to run a batch. Conversely, in a high-throughput surveillance setting, for which time to result is not crucial and samples can be stored and then run in larger batches, the lower-throughput iSeq100 will be less ideal. Looking at cost trajectories for WGS over large total numbers of samples processed and taking into account the cost of the sequencer, the MiSeq becomes more cost efficient over approximately 1,000 samples being processed (Fig 3), not including costs for personnel time or instrument maintenance.

For successful sequencing uptake in LMICs, there needs to be simplification of both the library preparation and the sequencer itself, which is crucial for a more decentralized solution. The development of an easier-to-use sequencer that requires less maintenance and less hands-on time than current instruments sets the stage for successful adoption of sequencing approaches in LMIC settings. The iSeq100 onboard pool denaturation reduces the number and complexity of hands-on steps. By having all the fluidics contained in the disposable cartridge, the complexity of run-to-run contamination is eliminated while the maintenance requirement is also reduced (Table 3). The iSeq100’s reduction in the complexity and number of hands-on steps for starting a sequencing run (Table 3) opens up sequencing as a feasible approach at a lower healthcare level compared to where current Illumina sequencing systems can be placed today. Additionally, the simplified initial setup and the reduced maintenance needs for the iSeq100 (Table 3) enable sequencing in a broader set of laboratories.

There were several limitations to this study. One limitation was that we conducted the targeted NGS portion of the study with a prototype cartridge on the iSeq100. This version of the cartridge allowed the addition of the required sequencing primers not found in the Illumina reagent cartridge. The commercially released reagent cartridge does not currently support this addition of primers. Another limitation is that we used custom sequencing primers that have not been optimized for the iSeq100 platform; to understand and address the errors associated with read 2, additional optimization of the custom sequencing primers will be needed. We felt this did not significantly affect our conclusions, however, as sufficient data were generated from read 1 to show a high level of agreement with the MiSeq. Another limitation was not optimizing sequencing pool loading concentrations. Further work will need to optimize sequencing pool loading concentrations specific to *M*. *tuberculosis* WGS libraries and our specific targeted NGS assay. As sequencing pool loading concentrations should be optimized to the GC content and genome size of the samples, the optimal cluster density would be achieved, thus helping to reduce background noise and increase the amount of usable sequence data generated for each sample.

In conclusion, for sequencing to have a significant and sustained impact on *M*. *tuberculosis* drug resistance diagnosis, pathogen surveillance, and precision medicine in LMICs, there is a real need for simplification of the sequencing process. The iSeq100 sequencer has comparable performance to the MiSeq sequencer with the benefit of sequencing in smaller batch sizes at an affordable price, combined with improved ease of use and reduced maintenance of the instrument. Future work will need to address the optimization of protocols to maximize data output for *M*. *tuberculosis* on iSeq100. Additionally, to utilize the targeted NGS method used in this study, the release of reagent cartridges that allow for user-supplied sequencing primers is needed.

## Supporting information

S1 Table WGS variant call percent agreement. WGS, whole-genome sequencing.
(DOCX)

S1 Text Prospective analysis plan. (DOCX)

S2 Text Clarification of Illumina’s role and contribution to project. (PDF)

## Abbreviations

AMK: amikacin
CAP: capreomycin
DR-TB: drug-resistant TB
DST: drug susceptibility testing
GC: guanine-cytosine
IGM: Institute for Genomic Medicine
INH: isoniazid
IVD: in vitro diagnostics
KAN: kanamycin
LMICs: low- and middle-income countries
MOX: moxifloxacin
NGS: next-generation sequencing
OFX: ofloxacin
RIF: rifampin
TB: tuberculosis
UCSD: University of California, San Diego
UVP: unified variant pipeline
WGS: whole-genome sequencing
