# Correction: Whole-genome and targeted sequencing of drug-resistant *Mycobacterium tuberculosis* on the iSeq100 and MiSeq: A performance, ease-of-use, and cost evaluation

**DOI:** 10.1371/journal.pmed.1002823

**Published:** 2019-06-03

**Authors:** The PLOS Medicine Staff

Following the inclusion of S2 Text in the original article to disclose the role of the funder Illumina in this study, the financial disclosure was not updated to reflect the same information. The Funding should read:

REC, CMD, TCR, and AM had funding from the Department for International Development from the United Kingdom award (204074–101) and Department of Foreign Affairs and Trade Australian Government award (70957). TCR had funding from National Institute of Allergy and Infectious Diseases (grant number: R21AI135756). MS was supported from the National Heart, Lung, and Blood Institute at the National Institutes of Health (grant number: T32HL134632). Illumina supplied early-access iSeq100 sequencing reagents; some experiments were conducted at Illumina by Illumina employees; the role of the funder Illumina is further described in S2 Text. The other funders had no role in study design, data collection and analysis, decision to publish, or preparation of the manuscript.
