# Supplementary material for: Whole-genome and targeted sequencing of drug-resistant Mycobacterium tuberculosis on the iSeq100 and MiSeq: A performance, ease-of-use, and cost evaluation
Source: PLoS Med. 2019 Apr 30;16(4):e1002794. doi: 10.1371/journal.pmed.1002794 (PMC6490892; doi:10.1371/journal.pmed.1002794)
Supplement: S1 Table — WGS, whole-genome sequencing. (DOCX) [file pmed.1002794.s001.docx]

S1 Table. WGS variant calls percent agreement

| Sample ID | MiSeq - UCSD/ MiSeq - Illumina | MiSeq - UCSD/ iSeq100 | MiSeq - Illumina/ iSeq |
| --- | --- | --- | --- |
| 20112 | 95.2 | 91.9 | 91.1 |
| 20066 | 97.4 | 96.2 | 95.3 |
| 20027 | 96.7 | 93.1 | 93.4 |
| Meta percent agreement  [CI.] | 97.6%  [97%-98.1%] | 94%  [93.1%-94.8%] | 93.6%  [92.6%-94.4%] |
