# Supplementary material for: Whole-genome and targeted sequencing of drug-resistant Mycobacterium tuberculosis on the iSeq100 and MiSeq: A performance, ease-of-use, and cost evaluation
Source: PLoS Med. 2019 Apr 30;16(4):e1002794. doi: 10.1371/journal.pmed.1002794 (PMC6490892; doi:10.1371/journal.pmed.1002794)
Supplement: S1 Text — (DOCX) [file pmed.1002794.s002.docx]

**PROJECT PLAN**

**Proof-of-Concept Study: iSeq 100 for Tuberculosis Surveillance Testing**

**Background:**

Next generation sequencing (“NGS”) is a technology of choice to build a standardized and validated variant detection (antimicrobial resistance) pipeline for *Mycobacterium tuberculosis* (TB). Methods to capture antibiotic resistance in TB are either *targeted* (amplicon based) or *whole genome* sequence based and have been utilizing the Illumina MiSeq NGS platform.

**Goals:**

This proof-of-concept study will test both methods on Illumina’s new iSeq 100 instrument in comparison to MiSeq runs. Comparative measures will include the specificity of detecting known mutations and the sensitivity for detecting hetero resistance.

**Study:**

1. *Targeted Method*

**UCSD Material:**

- 50 TB DNA samples from clinical isolates of diverse genetic composition
- 6 mixtures to mimic hetero resistance:

Mixtures of susceptible and resistant strains (XDR strains) at concentrations of 0.1, 1, 2, 5, 10 and 50% resistance

- 4 MTB negative sputum samples spiked with TB DNA

A total of 60 samples will be evaluating under this Project Plan.

**Illumina Consumables:**

- 2 MiSeq Reagent Kits v3 (600-cycle) – MS-102-3003
- 6 iSeq 100 i1 reagent kits - 20021533

**Research Plan:**

- UCSD will amplify regions in the TB genome which infer antimicrobial resistance and prepare libraries for a total of 60 samples including TB XDR samples, mixtures, and spiked sputum samples.
- UCSD will quantify and pool libraries for iSeq and MiSeq sequencing runs
- UCSD will provide library pool concentrations, sample names, and index sequences per sample to Illumina
- UCSD will sequence libraries on the MiSeq at the UCSD Core using a 2x300 read length
- UCSD will ship pooled, non-denatured libraries and Miseq 600 cycle reagent kit to Illumina to run them on MiSeq with 2x300 read length and iSeq 100 with 2x150 read length. (details below)

UCSD – 1 MiSeq run

MiSeq Library Pool

Targeted libraries - 60 samples/run - Illumina – 1 MiSeq run

iSeq 100 Library Pool Illumina – 6 iSeq 100 runs (1 duplicate)

~ 14 samples/run -

Illumina will run the pooled libraries on the MiSeq and the iSeq 100. All library pools will be spiked with ~25% PhiX control library prior to sequencing at each site. One iSeq 100 run will be performed in duplicate for reproducibility. iSeq 100 sequencing runs maybe performed on prototype or production-equivalent instruments with development or production-equivalent sequencing reagents. Illumina will provide sequencing instrument ID information to UCSD.

Illumina will perform demultiplexing and FASTQ file generation for MiSeq and iSeq 100 sequencing runs (which constitute the Illumina Results). Primary analysis of iSeq 100 sequencing runs maybe performed with development or production-equivalent versions of software. Illumina will provide per sample FASTQ files and tracking information to UCSD via BaseSpace. Data shall not be retained by Illumina UCSD may share the Illumina Results with FIND for co-authored publication (UCSD, FIND, Illumina).

1. *Whole Genome Sequencing Method*

**UCSD Material:**

- TB DNA samples from 3 clinical strains of diverse genetic composition
- Optional: Cultures of the same 3 strains

A total of 3-6 samples

A total of 6-9 libraries (3 Nextera XT libraries from DNA, 3 Nextera DNA Flex libraries from DNA, optional – 3 Nextera DNA Flex libraries from culture)

**Illumina Consumables:**

- 1 Nextera XT DNA Library Preparation Kit (24 samples) - FC-131-1024
- 1 Nextera XT Index Kit (24 indexes, 96 samples)) – FC-131-1001
- 1 Nextera DNA Flex Library Prep (24 samples) – 20018704
- 1 Nextera DNA CD Indexes (24 Indexes, 24 samples) – 20018707
- 2 MiSeq Reagent Kits v3 (600-cycle) – MS-102-3003

2-3 iSeq 100 i1 reagent kits - 20021533

**Research Plan:**

- UCSD will prepare libraries for small whole genome sequencing. Libraries will be prepared by UCSD from DNA using the Illumina Nextera XT kit and the Illumina Nextera DNA Flex kit per the Illumina User Guide protocol. Depending upon availability libraries will be prepared directly from culture of the same samples using the Illumina Nextera DNA Flex kit. Illumina will provide troubleshooting support if needed.
- UCSD will provide feedback to Illumina regarding performance and usability of Nextera DNA Flex kit. If any UCSD performs any deviations from the User Guide protocol for the library preparation, they will be noted and communicated to Illumina.
- UCSD will quantify and pool libraries for iSeq and MiSeq sequencing runs
- UCSD will provide library pool concentrations, sample names, and index sequences per sample to Illumina
- UCSD will sequence libraries on the MiSeq at the UCSD Core using a 2x300 read length
- UCSD will ship pooled, non-denatured libraries and Miseq 600 cycle reagent kit to Illumina to run them on MiSeq with 2x300 read length and iSeq 100 with 2x150 read length. (details below)

UCSD – 1 MiSeq run

MiSeq Library Pool

Targeted libraries - 6-9 samples/run - Illumina – 1 MiSeq run

iSeq 100 Library Pool Illumina – 2-3 iSeq 100 runs

- 3 samples/run -

Illumina will run the pooled libraries on the MiSeq and the iSeq 100. All library pools will be spiked with ~5% PhiX control library prior to sequencing at each site. One iSeq 100 run will be performed in duplicate for reproducibility. iSeq 100 sequencing runs maybe performed on prototype or production-equivalent instruments with development or production-equivalent sequencing reagents. Illumina will provide sequencing instrument ID information to UCSD.

Illumina will perform demultiplexing and FASTQ file generation for MiSeq and iSeq 100 sequencing runs (which constitute the Illumina Results). Primary analysis of iSeq 100 sequencing runs maybe performed with development or production-equivalent versions of software. Illumina will provide per sample FASTQ files and tracking information to UCSD via BaseSpace. Data shall not be retained by Illumina UCSD may share the Illumina Results with FIND for co-authored publication (UCSD, FIND, Illumina).
