# Supplementary material for: Whole-genome and targeted sequencing of drug-resistant Mycobacterium tuberculosis on the iSeq100 and MiSeq: A performance, ease-of-use, and cost evaluation
Source: PLoS Med. 2019 Apr 30;16(4):e1002794. doi: 10.1371/journal.pmed.1002794 (PMC6490892; doi:10.1371/journal.pmed.1002794)
Supplement: S2 Text — (PDF) [file pmed.1002794.s003.pdf]

S2 Text. Illumina's role and contribution to project

FIND and UCSD were solely responsible for the conceptualization and design of the study and were solely responsible for drafting the manuscript and making final decisions regarding the data, analyses and manuscript language.

The authors from Illumina (JH and AY) played a role in defining some of the specific sequencing methods as the iSeq100 sequencing instrument and reagents were only available in "early access" versions and only on the Illumina campus in San Diego, as the instrument was not yet available publically or commercially. Where specific methods and specifications of the new iSeq100 platform were needed to plan the study, JH and AY provided the information. For example, the amount of expected data to be output from the new iSeq100 instrument was provided by Illumina. We needed this information to develop batching and run parameters for the project.

As the platform was early access and not available to be run on the UCSD campus, Illumina also had to run the sequencer and record the raw data collected on the iSeq and Miseq instrument runs at Illumina. Additional Miseq sequencing was also run at UCSD to allow for comparison of the samples run at Illumina versus UCSD. At Illumina, co-author JH conducted the sequencing on the iSeq100, with oversight by AY. All data generated by Illumina was transferred to FIND and UCSD for analysis for the manuscript. Some initial data analysis by Illumina was required to determine the correct loading concentrations reported in the manuscript. For any analysis conducted by Illumina, FIND also conducted the same analysis with the transferred data to confirm findings. FIND and UCSD drafted the manuscript. The draft manuscript was shared with AY and JH to review and suggest edits as per the publication terms outlined in the FIND, UCSD, and Illumina 3-way material transfer agreement (MTA). FIND and UCSD had final say on any and all changes to the manuscript.
